# PROFILES OF PERSONAL DETERMINANTS OF HEALTH AND ASSOCIATED HEALTH OUTCOMES IN OLDER ADULTS: A LATENT CLASS ANALYSIS

**DOI:** 10.1093/geroni/igac059.2405

**Published:** 2022-12-20

**Authors:** Melanie Serrao Hill, Timothy Barnes, Lizi Wu, Rifky Tkatch, Sandra Kraemer, James Schaeffer, Charlotte Yeh

**Affiliations:** Optum Labs, Minnetonka, Minnesota, United States; Optum Labs, Minnetonka, Minnesota, United States; Optum Labs, Minnetonka, Minnesota, United States; Optum Labs, Minnetonka, Minnesota, United States; UnitedHealthcare, Minnetonka, Minnesota, United States; Optum Labs, Minnetonka, Minnesota, United States; ASI, Inc., Washington, District of Columbia, United States

## Abstract

Personal Determinants of Health (PDOH) are considered personal resources that contribute to successful aging. Positive PDOH factors have been associated with better health outcomes in older adults; however, research in this area is still limited. Using survey and claims data among adults age 65+ (N=2,866), profiles were identified based on five key PDOH factors (resilience, purpose in life, optimism, self-perception of aging, and social connection via loneliness) utilizing Latent Class Analysis (LCA). Differences in socio-demographics and healthcare outcomes were then explored utilizing multivariate regression models. Outcomes included emergency room (ER) visits, inpatient (IP) admissions, total medical cost, and quality of life (physical and mental health component scores). LCA models yielded three main classes within our sample: high PDOH (56%, n=1,150), moderate PDOH (40%, n=1,611), and low PDOH (4%, n=125). Those within the high PDOH class were significantly younger (Mage=75.6) compared to those within the moderate (Mage=78.2) and low (Mage=78.9) PDOH classes. In addition, the high PDOH class was significantly less likely to have an ER visit (OR=1.81, 95% CI:1.23-2.66) and had 28% better physical health and 52% better mental health components scores as compared to the low PDOH class. Results demonstrate support for the PDOH model as a potential successful aging model. Combinations used to create classes using resilience, purpose in life, optimism, self-perception of aging, and social connection via loneliness indicates that those who are positive in all five aspects show the most promising health outcomes. Future implications include targeting interventions at improving these variables for at-risk older adults.

